# An Experimental Evaluation of Competing Age-Predictions of Future Time Perspective between Workplace and Retirement Domains

**DOI:** 10.3389/fpsyg.2017.02316

**Published:** 2018-01-09

**Authors:** Matthew J. Kerry, Susan E. Embretson

**Affiliations:** ^1^Swiss Federal Institute of Technology, Zurich, Switzerland; ^2^Quantitative Psychology, Georgia Institute of Technology, Atlanta, GA, United States

**Keywords:** subjective life expectancy (SLE), future time perspective (FTP), experiment, rival hypotheses, theory elaboration

## Abstract

Future time perspective (FTP) is defined as “perceptions of the future as being limited or open-ended” (Lang and Carstensen, 2002; p. 125). The construct figures prominently in both workplace and retirement domains, but the age-predictions are competing: Workplace research predicts decreasing FTP age-change, in contrast, retirement scholars predict increasing FTP age-change. For the first time, these competing predictions are pitted in an experimental manipulation of subjective life expectancy (SLE). A sample of *N* = 207 older adults (age 45–60) working full-time (>30-h/week) were randomly assigned to SLE questions framed as either ‘Live-to’ or ‘Die-by’ to evaluate competing predictions for FTP. Results indicate general support for decreasing age-change in FTP, indicated by independent-sample *t*-tests showing lower FTP in the ‘Die-by’ framing condition. Further general-linear model analyses were conducted to test for interaction effects of retirement planning with experimental framings on FTP and intended retirement; While retirement planning buffered FTP’s decrease, simple-effects also revealed that retirement planning increased intentions for sooner retirement, but lack of planning increased intentions for later retirement. Discussion centers on practical implications of our findings and consequences validity evidence in future empirical research of FTP in both workplace and retirement domains.

## Introduction

Because of longer life expectancies, adults are working or retiring longer, or both (Ilmarinen, 2005). In contrast, foreshortened life expectancies reduce employment or retirement experience. Which phase is impacted, however, is likely influenced by one’s motivation to work longer or not. To date, empirical evidence has indicated positive effects of age on both, retirement planning and retirement intentions, i.e., later retirement (Montalto et al., 2000). Functionally, workplace and retirement scholars similarly locate future time perspective (FTP), defined as “perceptions of the future as being limited or open-ended” (Lang and Carstensen, 2002; p. 125), as a mediator between age and respective focal criteria. For example, workplace researchers regard FTP as mediating age effects for retirement intentions (RetirIntent; Henry et al., 2017). Consequently, workplace researchers suggest workplace solutions may reduce FTP’s decline and, thereby, retain older knowledge workers by *prolonging* retirement intentions. On the other hand, retirement scholars regard FTP as mediating age effects for retirement planning (RetirPlan; Hershey and Mowen, 2000)^1^. Consequently, retirement researchers suggest mindfulness solutions may strengthen FTP’s increase and, thereby, prompt earlier retirement planning by accelerating preparations for wellbeing in retirement.

**FIGURE 1:**
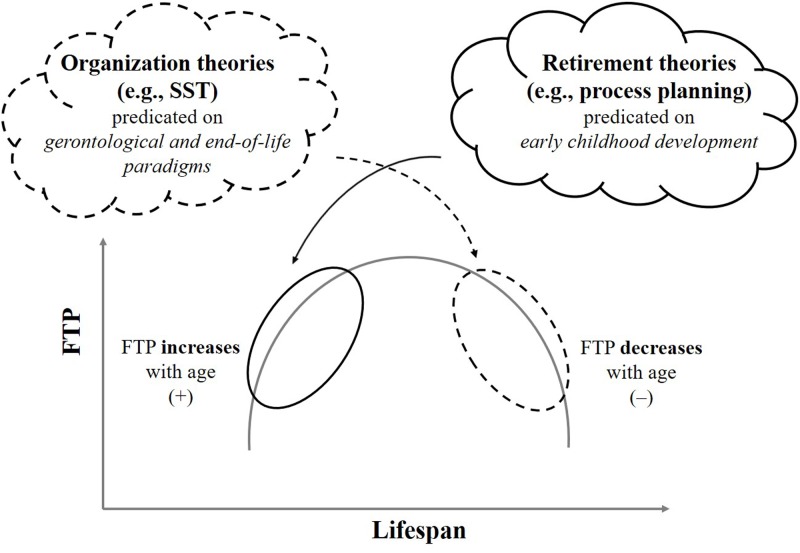
Lifespan theoretic-disciplinary origins for contradictory age-related predicts for FTP. FTP, future time perspective; SST, socioemotional selectivity theory.

The predicted directions of the FTP-mechanism with age, however, are competing, such that retirement scholars predict increasing FTP with age (Hershey and Mowen, 2000), whereas workplace scholars predict decreasing FTP with age (Griffin et al., 2012), consistent with inveterate lifespan theories (Carstensen and Lang, 1996, Unpublished). Effectively, the rival hypotheses can be understood from two valid perspectives on workforce aging, (1) Relative aging (as cohort effect), and (2) Absolute aging (as longer life expectancies). Although both perspectives are reasonable, the implication from ‘absolute’ aging is that chronological age’s explanatory value for developmental predictions will deteriorate continuously (proxy indicator detaches further from longevity complexions). This is evidenced by the fact that absolute aging (work-life and post-work life) will likely continue after the relative aging (graying from Baby Boomer cohort) subsides. Consequently, the substantive value of age-related predictions in both workplace and retirement researches is depreciating. Causally, however, **Figure 1** displays the disciplinary sources for the competing age-related predictions for FTP. To date, the competing predictions have not been evaluated (c.f., see Rudolph et al., 2017). As Wang and Schultz (2010, p. 176) described the disjunction between workplace and retirement fields of research, “…very few studies that examined outcomes of retirement have incorporated factors that influenced the original retirement decision…This creates a logic gap because the reasons why people decide to retire would naturally influence how they evaluate outcomes.”

**Figure 1** is a good example of how narrow disciplines and misinterpretation of external correlates for construct meaning (rather than internal validity sources) converge to yield static domain structures. That is, conditioning predictions on age for meaningful interpretation of relevant criteria across workplace and retirement domains, requires independence assumptions between work and retirement contexts. Independence of work – retirement domains is intuitively flawed, given the dependence of retirement from a previous worklife. Consequently, age is limitedly meaningful for the growing complexity and overlap of work and retirement contexts. This practical disconnect evidences in probabilistically opposing (directionally competing) age-predictions for FTP concerning the focal criteria of interest (delaying intentions/advancing preparations for retirement).

In contrast to age effects on employment and retirement, much less research attention has been given to its counterpoint, that is, subjective life expectancy (SLE; Griffin et al., 2013). Empirical evidence has indicated that perceptions of remaining time in later stages of life are comparatively better predictors than age for decision processes and transitions, e.g., retirement, (van Solinge and Henkens, 2010). Interestingly, workplace and retirement scholars locate FTP in similar nomological networks vis-à-vis intended retirement (**Table 1**).

**Table 1:** Summary of directional empirical findings.

Antecedents	Mechanisms	Consequences
Age	(+) Retirement planning	(-) Intended retirement
Age	(+/-) FTP	(+) Intended retirement
SLE	(?) Retirement planning	(-) Intended retirement
SLE	(?) FTP	(+) Intended retirement

Taken together, current empirical findings suggest that RetirPlan (accelerator) and FTP (delayer) may operate as competing mechanisms on retirement decisions Empirical support for the first mechanism, RetirPlan as-accelerator of workforce withdrawal is well-illustrated by the design of Thaler and Benartzi’s (2004) ‘Save for Tomorrow’ United States retirement pension program. Its dynamic feature, to auto-increase savings contributions over employment, is premised on counteracting increasing discount rate (and retirement delays) as a function of aging. This reasoning is further consistent with recent empirical findings that substantively distinguishes between temporal quantification and domain-specific goal strivings as mechanisms to specific facets of developmental self-regulation (Kornadt et al., 2017).

Regarding the second mechanism, FTP as-delayer of retirement intentions, it has received empirical support from a study that indicated, future-oriented prospections induced escalations-of-commitment (i.e., sunk-cost biases; Strough et al., 2016). Additional, indirect evidence from a meta-analysis of FTP and procrastination reported a moderate-negative association of *r* = -0.45 (Sirois, 2014). Moreover, mediation tests of perceived stress and positive-affect suggested that positive-affect operates as a significant-mediator between FTP and procrastination; Inclusion of the mediator resulted in the unconditional-direct effect of FTP-procrastination to switch signs (from negative to positive).

Retention of older workers, by aligning workplace conditions with growing ‘stability preferences’ is premised on counteracting the opportunity cost from workforce continuation at older age; Indeed, workplace conditions typically govern worker wellbeing, and poor quality workplaces render their occupants decremented in wellbeing to levels comparable to, both the unemployed and disabled (ten Have et al., 2015). Extrapolating, a systematic review of the association between the latest economic crisis (i.e., low job mobility opportunity) and workplace stressors found that job insecurity was the strongest correlate with mental health impairments (Mucci et al., 2016).

Synthesizing conceptions across empirical studies, the impacts of: (1) work vs. non-work valuation (affect), and (2) short vs. long time horizons (cognitive), as two mechanisms linking work and retirement has not yet been evaluated in a single study. The goal of the current experiment, therefore, is to pit these competing mechanisms in an evaluative framework by manipulating SLE via attribute-framing conditions. The results are expected to clarify competing age-predictions for FTP across workplace and retirement research domains. Next, we will introduce our SLE manipulation for subsequently deriving our research hypotheses.

### Hypotheses

The current study evaluates the competing predictions for age-FTP with an attribute-framing manipulation of SLE and simultaneous estimation of FTP and RetirIntent. Recent findings indicate that the framing of SLE questions, as either ‘Live-to’ or ‘Die-by,’ results in an SLE difference of approximately 7–10 years (Payne et al., 2013). Examples of the ‘Live-to’ and ‘Die-by’ framings are “What is the probability that you currently expect to ‘**live-to**’/‘**die-by**’ 95-years of age? The ‘Die-by’ frame has been found to lower SLE compared to ‘Live-to’ framings.

Conceptually, we may re-interpret this SLE manipulation as a ‘subjective age’ manipulation. In this case, if the ‘Die-by’ frame is successfully replicated so that it lowers SLE, we may interpret this as inducing ‘older subjective age.’ Consequently, the randomization of participants and comparison on FTP enables us to infer how FTP covaries with different perceptions of one’s remaining lifetime/SLE. Restated in the context of formal hypothesis-testing, we hypothesize that the ‘Die-by’ SLE frame will reduce FTP compared to the ‘Live-to’ frame.

H1: The ‘Die-by’ frame will decrease FTP compared to the ‘Live-to’ frame.

Additionally, we include the variable ‘retirement intention’ in order to assess the consistency of directional effects across ‘work’ and ‘retirement’ domains. Specifically, if we accept retirement to be subsumed by overall age, then it follows that older workers (lower SLE from ‘die-by’ frame) should report closer proximity to retirement (sooner retirement). Thus, we also hypothesize that the ‘Die-by’ SLE frame will reduce retirement intentions compared to the ‘Live-to’ frame.

H2: The ‘Die-by’ frame will decrease RetirIntent compared to ‘Live-to’ frame.

Finally, ‘retirement planning’ is purposefully assessed before randomized treatment assignment (live-to/die-by). Therefore, because ‘retirement planning’ is domain-specific to ‘life after retirement,’ we assume that greater planning for this post-work time will reduce the negative effect of lowered SLE on FTP as a ‘global’ construct. That is, we hypothesize that RetirPlan will operate by interacting with the ‘Die-by’ frame by buffering its negative effect on FTP.

H3: RetirPlan will interact with the ‘Die-by’ frame on FTP, such that high RetirPlan will reduce the negative effect.

In contrast, we expect that the domain-specificity of ‘retirement planning’ and ‘retirement intention’ to conjoin, so that the hypothesized main-effect of ‘sooner retirement’ will be strengthened by more retirement planning (assuming planning is proximal of this post-work life). That is, we hypothesize that RetirPlan will operate by interacting with the ‘Die-by’ frame by strengthening its negative effect on retirement intentions.

H4: RetirPlan will interact with the ‘Die-by’ frame on RetirIntent, such that high RetirPlan will strengthen the negative effect.

In addition to hypothesizing SLE-manipulation effects via response processes, that is, on mean-level changes of our focal outcomes, we further explore the nomological pattern of correlates with relevant external variables, in particular, the association between RetirPlan and RetirIntent.

## Materials and Methods

A sample of *N* = 207 participated in ‘2 × 1’ between-subjects experimental design. SLE served as between-subjects factor via the framing manipulation (‘Live-to’ vs. ‘Die-by’). Inclusion criteria required that participants be presently working full-time (>30-h/week) for appropriate study of focal retirement variables. Amazon’s Mechanical Turks crowdsourcing labor market was used for participant recruitment. Participants received nominal compensation ($1.50) for completion of the online experiment-questionnaire, with an additional incentive offered via a $100 lottery drawing.

### Ethical Considerations

**FIGURE 2:**
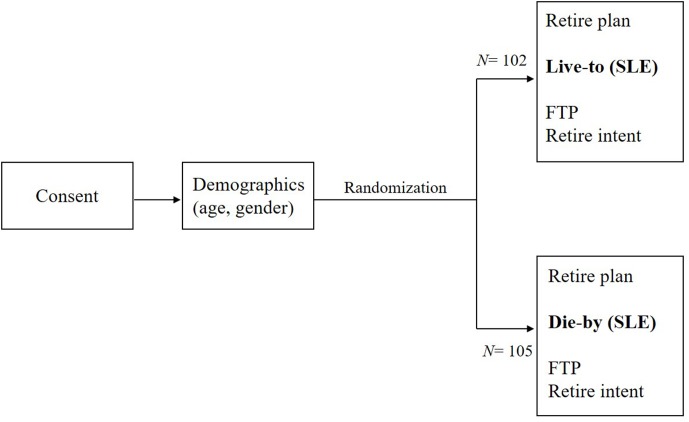
Participant flow through online survey protocol. SLE, subjective life expectancy; FTP, future time perspective.

This study was carried out in accordance with the recommendations of ‘international-ethical human study guidelines’ in accordance with the Declaration of Helsinki. The protocol was approved by the authors’ academic institutional review board (Georgia Institute of Technology). All subjects were given written, informed consent prior to participating in the online questionnaire. Additional educational materials on retirement preparation was provided for interested participants on completion of the questionnaire. See, **Figure 2** below.

### Measures

#### Subjective Life Expectancy

Subjective life expectancy was assessed on a slider-scale with probabilities ranging from 0 to 100. Participants were instructed to use the slider to indicate their probability of living to ages 65, 75, 85, and 95-years old. Instructions varied with experimental condition, so that participants assigned to the ‘live-to’/‘die-by’ condition read, “Using the slider scale below, please indicate the likelihood you currently believe you will ‘live-to’/‘die-by’ 65 / 75 / 85 / 95-years of age. Cronbach’s internal reliability was estimated at α = 0.83.

#### Future Time Perspective

Carstensen and Lang’s (1996, unpublished) original 10-item instrument was administered to assess FTP^2^. Participants indicated their agreement with how “true” each item described themselves using a 7-point Likert-type scale from 1 (Very untrue) to 7 (Very true). A sample item is, “(1) Many opportunities await me in the future.” Cronbach’s internal reliability was estimated at α = 0.91.

#### Retirement Intentions

RetirIntent was assessed as a four-item composite of intended retirement age across four domains: ‘current employer,’ ‘career,’ ‘occupation,’ and ‘general workforce.’ Responses were recorded on a continuous scale. A sample item is, “I intend to retire at age 66–95.” Cronbach’s internal reliability was estimated at α = 0.89.

#### Retirement Planning

RetirPlan was assessed with Griffin and Hesketh’s (2008) 11-item global measure of planning activities. Participants indicated their behavioral tendency to engage in preparatory activities on a 5-point Likert-type scale from 1 (Not at all) to 5 (A great deal). A sample item is, “Seeking retirement advice about retirement issues.” Cronbach’s internal reliability was estimated at α = 0.90.

### Analyses

Preliminary response-quality screening, missing-data assessment, and hypotheses testing was conducted in SPSS v23. To test main-effects across our randomly allocated experimental groups, independent-sample *t*-tests were conducted to compare means on FTP and RetireIntent. High- and low-RetirPlan groups were generated with median-splits on the RetirPlan variable in order to test interaction-hypotheses in our experimental design. In order to test for interaction-effect hypotheses, SPSS’s univariate-general linear model procedures were implemented with SLE-experimental condition (Live-to vs. Die-by) and RetPlan groups (high vs. low) entered in a full-factorial model. Type III sums-of-squares were utilized in parameter estimate calculations. Significant-interactions were further inspected with simple pairwise-comparisons of experimental conditions within each level of the data-driven moderator (low/high RetirPlan). Bonferroni’s corrections were implemented for managing Type-1 error rates from multiple-comparison tests. The observed-score general linear model was selected over latent-variable approaches^3^ due to its parsimony, including lower sample size requirements and our focus on competing predictions between specific relations/localized pathways (compared to latent-variables’ usage for overall-model comparisons).

## Results

Summary sample characteristics are reported in **Table 2** below. SLE-manipulation effects were successfully replicated, such that participants in the ‘Live-to’ condition reported a 10.02% greater chance of living to age 80 compared to the ‘Die-by’ condition [*t*_(205)_ = 3.75, *p* < 0.01, Cohen’s *d* = 0.52]. Notably, we observed a differential correlation between SLE-FTP that is consistent with theoretically competing age-predictions for FTP, *r* = 0.40_(Live)_**/**-0.30_(Die)_. That is, the directional discrepancy may be retraced to ‘younger’ and ‘older’ populations that were the foci for early-child development and gerontological theories predating the lifespan framework’s practical integration.

**Table 2:** Summary sample characteristics by experimental conditions.

	Live-to	Die-by
Size (*N*)	102	105
Age M (*SD*)	57.1 (3.84)	57.0 (4.40)
Gender
Male	50%	50%
Female	50%	50%
SLE	76.59 (1.75)	73.92 (1.67)

*Age is reported as sample mean (M) with standard deviations (SD) in parentheses. Gender is reported as proportions (%). SLE = subjective life expectancy. Independent-sample t-tests were not-significant (*p* > 0.05) for demographics and moderator variable RetirPlan, which supported assumptions of equivalence between the two groups*.

### Main Effects: Live-to vs. Die-by

Main effect hypotheses received mixed-support. Supporting H1, participants assigned to the ‘Die-by’ condition reported significantly lower FTP (*M* = 44.74, *SD* = 11.15) compared to the ‘Live-to’ condition (*M* = 47.92, *SD* = 13.14); *t*(205) = 3.44, *p* = 0.03, Cohen’s *d* = 0.48.

**FIGURE 3:**
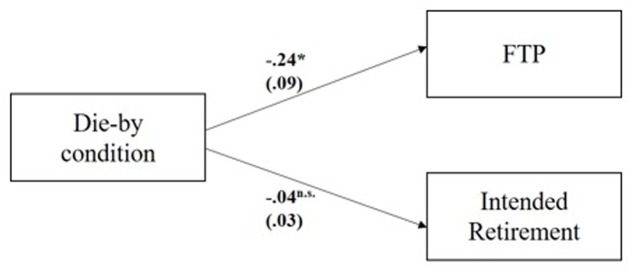
Main effect of die-by condition on mean FTP and intended retirement. Standardized mean differences reported as path estimates with 95% standardized errors displayed in parenthesis; *N* = 206; FTP, future time perspective.

For H2, participants assigned to the ‘Die-by’ condition also reported lower RetirIntent (*M* = 2.08, *SD* = 1.06) compared to the ‘Live-to’ condition (*M* = 2.04, *SD* = 1.14), but the effect was not significant; *t*(205) = 0.52, *p* = 0.31, Cohen’s *d* = 0.07. Main effect results are summarized in **Figure 3** below as standardized reductions of the ‘Die-by’ frame compared to the ‘Live-to’ frame.

### Interaction Effects

**FIGURE 4:**
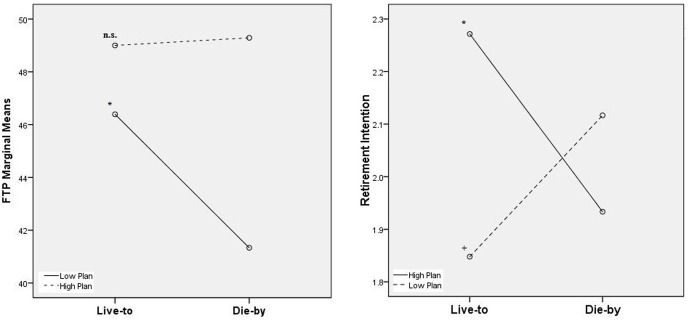
Experimental marginal-means of FTP (left) and retirement intention (right) by High/Low planners. ^n.s^non-significant experimental effect (*F* = 0.01,*p* = 0.96). ^∗^*p* < 0.05 (*F* = 4.27[left]/*F* = 4.11[right]). **^+^***p* < 0.10 (F=2.72). Degrees of freedom between, within = 1,203 for all simple-contrasts.

Interaction hypotheses for RetirPlan were generally supported. Specifically, regarding H3 for RetirPlan’s buffering effect of the ‘Die-by’ frame on FTP, results indicated a significant interaction, *F*(1,203) = 3.01, *p* = 0.04^4^. A simple-contrast estimate indicated significance in the hypothesized direction, such that the ‘Die-by’ frame’s negative effect on FTP was limited to low RetirPlan [*F*(1,203) = 4.27, *p* = 0.02]. In contrast, for high RetirPlan, the ‘Die-by’ frame had no effect on FTP [*F*(1,203) = 0.01, *p* = 0.96]. FTP’s marginal-means used for simple-contrasts are illustrated in the left-panel of **Figure 4**.

Regarding H4 for RetirPlan’s enhancing effect of the ‘Die-by’ frame on RetirIntent, results indicated a significant interaction, *F*(1,203) = 3.40, *p* = 0.03. A simple-contrast estimate indicated significance in the hypothesized direction, such that the ‘Die-by’ frame’s negative effect on RetirIntent was significant for high RetirPlan [*F*(1,203) = 4.11, *p* = 0.04]. For low RetirPlan, an unexpected *increase* in RetirIntent was also observed, although it was only marginally significant [*F*(1,203) = 2.72, *p* = 0.10]. This unexpected cross-over interaction helps to explain the unobservable main-effect for RetirIntent, which would seem to-have been obscured. RetirIntent’s marginal means used for these calculations are illustrated in the right-panel of **Figure 4**.

Finally, **Table 3** below summarizes descriptive statistics for all simple-pairwise comparisons. Specifically, each row of **Table 3** lists the mean differences of all 2 × 2 combinations of low/high RetirPlan for FTP and RetirIntent with Bonferroni-adjusted 95% confidence intervals [CIs] reported.

**Table 3:** Pairwise-comparisons of experimental mean-differences for FTP and RetirIntent by Low/High plan groups.

Comparison by Low/High RetirPlan	Estimated mean difference	Standard error of difference	Bonferroni adjusted 95% CI
Low plan			
‘L-FTP’ – ‘D-FTP’	5.06^∗^	2.44	0.231, 9.89
High plan			
‘L-FTP’ – ‘D-FTP’	-0.29	2.50	-5.22, 4.64
Low plan			
‘L-RetIntent’ – ‘D-RetIntent’	0.27**^+^**	0.18	-0.13, 0.80
High plan
‘L-RetIntent’ – ‘D-RetIntent’	-0.39^∗^	0.16	-0.77, -0.01

^∗^*p < 0.05, ^+^*p* < 0.10, where *p*-values are adjusted using the Bonferroni method. Prefix ‘L’ denotes ‘Live-to’ experimental condition, prefix ‘D’ denotes ‘Die-by’ experimental condition*.

### Exploratory Analyses

In addition to mean-level effects on focal outcomes, the patterning of external correlates was different across ‘Live-to’ and ‘Die-by’ conditions. Specifically, for the ‘Live-to’ condition, a significant positive correlation was observed between RetirPlan-RetirIntent, *r* = 0.19, *p* = 0.04. In contrast, for the ‘Die-by’ condition, a significant negative correlation was observed, *r* = -0.22, *p* = 0.03. The latter finding supports views of RetirPlan as a mechanism that accelerates workforce withdrawal (earlier retirement).

Given the patterning of external correlates, we considered additional explanatory causes for our experimental findings to support substantive interpretation of RetirPlan’s moderation effects. First, we considered if the FTP main effect may be attributable to its sensitivity to ‘careless responding’ based on mix-scored items (positive- / reverse-scored). An independent-samples *t*-test was re-conducted, excluding the *j* = 3 reverse-scored FTP items. There remained a significant difference for the FTP subscore in the hypothesized direction across ‘Live-to’ (*M* = 33.11, *SD* = 8.33) and ‘Die-by’ (*M* = 30.69, *SD* = 9.67) conditions; *t*(205) = 3.84, *p* = 0.03, Cohen’s *d* = 0.54. The pattern of interactions also remained unchanged in terms of direction and statistical significance.

Because our RetirIntent outcome comprised no reverse-scored items, we examined if the non-significant main effect would hold across a median-split on survey duration (i.e., ‘time on test’). An independent-samples *t*-test was re-conducted separately for the low-Time and high-Time groups. The non-significant difference for RetirIntent remained for both the low-Time [*t*(101) = 0.46, *p* = 0.65, Cohen’s *d* = 0.06] and high-Time [*t*(103) = 0.85, *p* = 0.40, Cohen’s *d* = 0.12] groups. The pattern of interactions also remained unchanged in terms of direction and statistical significance. We summarize our findings and briefly elaborate their implications for future work-retirement integrative research in the section “Discussion” below.

## Discussion

The current study identified a construct, FTP, with disproportionate external validity evidence in a shifting applied context, specifically, workers and retirees. The mono-operation bias inherent to two contrarian theoretical origins of FTP across functionally dissimilar populations (workers and retirees), thus, vesseled weak validity evidence and poor verisimilitude (Cronbach, 1988). We evaluated the rival age-related hypotheses with an experimental SLE-framing manipulation.

Summarizing our findings, first, we successfully replicated the SLE-framing manipulation and observed the opposing relations between SLE-FTP, which was positive in the ‘Live-to’ condition (*r* = 0.40) and negative in the ‘Die-by’ condition (*r* = -0.30). For hypotheses, H1 was supported with a significant negative effect of the ‘Die-by’ condition on FTP. The finding generally support inveterate lifespan theories that postulate age-related decline in FTP (Carstensen and Lang, 1996, Unpublished). Furthermore, we received support for FTP’s hypothesized interaction with RetirPlan, such that participants reporting more RetirPlan were unaffected by the ‘Die-by’ framing’s negative impact on FTP.

On the other hand, H2 pertaining to RetirIntent was unsupported by our findings. There was no significant difference between SLE-frames for RetirIntent. Further inspection of the interaction hypothesis (H4) revealed an unexpected cross-over effect, providing some indication for obscuring the hypothesized main effect. Contrast analyses supported our hypothesis for RetirPlan’s strengthening of the negative ‘Die-by’ effect. On the other hand, we also observed an unanticipated positive effect of the ‘Die-by’ condition on RetirIntent for low-RetirPlan. The cross-over effect could be interpreted as a delay of RetirIntent when shorter SLE is combined with inadequate preparation. This interpretation is consistent with recent empirical findings that indicate FTP’s dual-mechanism of prompting planning and inducing sunk-cost biases/escalations of commitment with current conditions. That is, in the same way that job characteristics may enhance occupational FTP, contracted lifespans may induce avoidance of retirement’s unknown, if ill-prepared, event space.

Taken together, we hope that the current findings hold practical import for bettering the practice of career counseling and job crafting amid longer working lives. For example, practical implications of this experimental study intersect the age-integration of social institutions and domain-integration of work-life balance. To this end, results should inform ongoing areas of research on related lifespan concepts – For example, ‘active aging’ (World Health Organization, 2002), ‘aging-in-place’ (Center for Disease Control and Prevention, 2011), and ‘successful aging in the workplace’ (Krause, 2001).

### Limitations

We aimed to evaluate contradictory predictions for age-related changes in FTP. Although, we found support for negative age-related predictions for FTP, we also observed a substantive content responsitivity effect (Nichols et al., 1989). A tenable mechanism to explain such effects stem from empirical findings on mortality salience, specifically, its impact on identified determinants of motivated responding. For example, mortality salience is positively associated with prosocial attitudes (Jonas et al., 2002) and trust beliefs (Paulhus, 1983). The cognitive context of our SLE elicitations systematically impacted response processes, in turn, un-differentiable main effects were observed on RetirIntent. On the other hand, we observed an unexpected crossover interaction, such that participants in the ‘Die-by’ reporting low RetPlan reported significantly *later* RetirIntent. Although exploratory analyses were conducted to increase substantive inference from our findings, important limitations are noteworthy for cautious interpretation. For example, from a response-modeling perspective, our focus on latent-structural relations limited our examination of potential response biases to classical approaches, such as conditioning on standard- and reverse-scored items (Colsher and Wallace, 1989). More advanced item response theory (IRT) models, however, have recently been published, enabling researchers to precisely account for wording effects and model directly, any number of suspected response styles (e.g., midpoint, extreme, acquiescence; Falk and Cai, 2016). Second, regarding RetIntent, we presumed that our sample of older employees anticipated a future withdrawal from the workforce toward retirement. It is tenable, however, that low-RetPlan participants did not represent inadequate- or ill-preparation but, rather, a deliberate intention to never retire.

### Future Directions

The continually aging workforce puts a premium on organizational and retirement scholars’ specification of lifespan motivation theories (Löckenhoff, 2012). The psychological construct of FTP figures prominently in both areas of research; for work scholars, the socio-emotional selectivity theory (SST) of lifespan motivation predicts that FTP decreases with age (Carstensen and Lang, 1996, Unpublished). In opposition, retirement scholars employ a psycho-motivational model, adapted from a theory of lifespan planning that predicts FTP increases with age (Hershey, 2004).

Sophisticated IRT models offer explanatory testing of suspected response biases for self-report measures. For example, Kam and Meyer (2015) recently examined the broad implications of item valence (i.e., standard- and reverse-scoring) for, both factor loading-patterns and bivariate external correlates. Future research should continue to integrate cognitive context of the assessment scenario (including response instructions/item framing) with IRT models sensitive to potential response styles or construct distortion.

Regarding workplace adaptations of FTP, redesigning work to motivate older employees’ may not function as increase of occupational-FTP, rather, as a decrease in waiting costs, as Paglieri observes, “Costs of delay should be taken into account, on par with delay, in determining intertemporal choices. From this viewpoint, refusing to sustain the costs of a delay is not the same thing as devaluing a reward because it is delayed” (Paglieri, 2013, p. 371). A future experimental study examines this possibility (alternative mechanism) with a battery of decisional delay-discounting, psychophysical patience tests, and the FTP questionnaire (see also, Scheibe and Zacher, 2013). Summarizing, the new context of aging workforces and retirees presents an opportunity for theory elaboration in future research agendas (Fisher and Aguinis, 2017).

## Conclusion

The divide between work and retirement scholarship may be a reification of initial schisms between lifespan researches (early childhood vs. gerontological). The current authors concur with Shepard’s (1993) articulation of consequential validity regarding unintended consequences of measures, specifically, that it is a “logical extension” (p. 426) of Campbell’s (1960) advocacy for rival hypotheses in validity evaluations. Understanding of the aging labor force, by extension, requires a more comprehensive understanding of the motivations for retiring and for continuation. Should longer life expectancies abide the relative-cohort effect, then age-based predictions may depreciate in usefulness amid ever-longer and more complex employment lives. The contribution of the current experiment’s findings is that, not all work redesigns will encourage adequate planners to forego retirement, and not all who continue working past retirement are meaningfully present at work.

## Author Contributions

MK designed the experiment, recruited and ran participants, and conducted primary analyses. SE provided advanced-methodological consultancy and conducted expository write-up.

## Conflict of Interest Statement

The authors declare that the research was conducted in the absence of any commercial or financial relationships that could be construed as a potential conflict of interest.

## Footnotes

1 See Supplementary Appendix Table S1 of FTP-criteria correlates.

2 See Supplementary Appendix Table S2 for all FTP items.

3 Model-comparisons were also conducted with structural equation modeling (SEM) without substantive change to parameter estimates and nominal significance levels.

4 Similar analyses were conducted in an ordinary-least squares regression framework by computing a ‘Live/Die × RetirPlan’ multiplicative-interaction term. The equivalent results are reported here for complementary interpretation. Specifically, the ‘Live/Die × RetPlan’ regression-interaction coefficient was estimated at, *b* = 0.24, *p* = 0.04, supporting H3 and RetPlan’s buffering effect on FTP. Additionally, regarding H4 and RetirIntent, results indicated a significant interaction, such that the ‘Live/Die × RetPlan’ regression-interaction coefficient was estimated at, *b* = 0.26, *p* = 0.03.
