# Supplementary material for: An Experimental Evaluation of Competing Age-Predictions of Future Time Perspective between Workplace and Retirement Domains
Source: Front Psychol. 2018 Jan 9;8:2316. doi: 10.3389/fpsyg.2017.02316 (PMC5767307; doi:10.3389/fpsyg.2017.02316)
Supplement: Supplementary file 1 [file Data_Sheet_1.DOCX]

| Supplementary Appendix – Table S1  *Comparison of Two Future Time Perspective Questionnaires: Construct Conceptualization, Theoretical Postulates, and Item-Content.* | |
| --- | --- |
| **H-FTP** | **C-FTP** |
| **Construct Conceptualization and Definitions** | |
| Conceptualized as ‘central’ trait within personality hierarchy, between cardinal and surface traits (Buss, 1989).  Defined as, “patience or planning horizon…disproportional focus on future events” (Hershey, 2004, p. 34).  “Designed to tap the extent to which individuals enjoy thinking about and planning for the future” (Hershey, 2007, p. 30). | Conceptualized as malleable, cognitive-motivational construct.  Defined as, “perceptions of the future as being limited or open-ended” (Lang & Carstensen, 2002, p.125).  Defined as, “Perception of one’s future time as expansive and full of opportunities versus limited with few remaining opportunities” (Cate & John, 2007). |
| **Theoretical Postulates** | |
| FTP has *positive* relation with age. | FTP has *negative* relation with age. |
| **Item-Content** | |
| (Hershey & Mowen 2000) Future Time Orientation  1. I enjoy thinking about how I will live 10+ years in the future.  2. I have established long-term goals and am working to fulfill them.  3. It is very hard for me to visualize the kind of person I will be 10  years from now. (r)  4. The future seems very vague and uncertain to me. (r)  (Hershey et al. 2007) Future Time Perspective  1. I follow the advice to save for a rainy day.  2. I enjoy thinking how I will live years from now in the future.  3. The distant future is too uncertain to plan for. (r)  4. The future seems very vague and uncertain to me. (r)  5. I pretty much live on a day-to-day basis. (r)  6. I enjoy living for the moment and not knowing what tomorrow will bring. (r) | (Carstensen & Lang, 1996; Carsten 2006) Future Time Perspective   1. Many opportunities await me in the future 2. Most of my life still lies ahead of me 3. My future seems infinite to me 4. I expect that I will set many new goals in the future 5. My future is filled with possibilities 6. I could do anything I want in the future 7. There is plenty of time left in my life to make new plans 8. I have the sense that time is running out (r) 9. As I get older, I begin to experience time is limited (r) 10. There are only limited possibilities in my future (r) |
| *Note.* Items followed by (r) indicate reverse-scored items. *H-FTP* = Hershey future time perspective, *C-FTP* = Carstensen future time perspective. | |
