# Supplementary material for: An Experimental Evaluation of Competing Age-Predictions of Future Time Perspective between Workplace and Retirement Domains
Source: Front Psychol. 2018 Jan 9;8:2316. doi: 10.3389/fpsyg.2017.02316 (PMC5767307; doi:10.3389/fpsyg.2017.02316)
Supplement: Supplementary file 2 [file Data_Sheet_2.docx]

| Supplementary Appendix - Table S2  *Comparison of External Correlates of Two Future Time Perspective Questionnaires.* | | | | | | | |
| --- | --- | --- | --- | --- | --- | --- | --- |
| **H-FTP** | | | | **C-FTP** | | | |
| **Age** | | | | | | | |
| **Study** | **Sample (*N*)** | **Design** | **ES *r*** | **Study** | **Sample (*N*)** | **Design** | **ES *r*** |
| Hershey et al. (2007) | Full-time employees (*265*)  Age_M_ = 36.3 (6.18) [25-45] | X | -.03 | Schwall (2008) | Community adults (*N* = 233)  Age_M_ = 47 (10.6) | X | -.37** |
| Hershey & Mowen (2000) | Community adults (*230*)  Age_M_ = 62.6 (12.5) | X | -.01 | Schwall (2008) | Community adults (*N* = 387)  Age_M_ = 52 (7.2) [>40] | X | -.18** |
| Noone et al (2010)^a^ | Community adults (*N* = 1,532)  Age_M_ = 53.4 (2.89) [49-60] | X | .00 | Schwall (2008) | Community adults (*N* = 331)  Age_M_ = 47.2 (10.6) | X | -.36** |
| Hershey et al. (2010) | Community adults^f^ (*N* = 975)  Age_M_ = 42.5 (10.5) [25-65] | X | .07 | Schwall (2008) | Community adults (*N* = 368)  Age_M_ = 48.6 (10.3) | X | -.19** |
|  |  |  |  | Kessler & Staudinger (2011) | Community adults (*N* = 277)  Age_M_ = 47.5 (16.8) | X | -.67** |
|  |  |  |  | Lang & Carstensen (2002) | Community adults (*N* = 480)  Age_M_ = 55.7 (5.8) | X | -.70** |
|  |  |  |  | Treadway et al. (2012) | Full-time adults (*N* = 291)  Age_M_ = 30.6 (10.5) | X | -.35** |
|  |  |  |  | Kooij (2010) | University workers (*N* = 662)  Age_M_ = 44.2 (10.9) | X^b^ | -.67** |
|  |  |  |  | Bluck & Alea (2009)  Löckenhoff, O’Donoghue, & Dunning (2011) | Community adults (*N* = 185)  Age_M(Young)_ = 19.31 (2.80) Age_M(Old)_ = 73.04, (7.53)  Community adults (*N* = 98)  Age_M_ = 52.00 (20.5) | X^e^  X | -.63**  -.43** |
| **Retirement Planning** | | | | | | | |
| Table 3 (continued) | | | | | | | |
| **Study** | **Sample (*N*)** | **Design** | **ES *r*** | **Study** | **Sample (*N*)** | **Design** | **ES *r*** |
| Hershey et al. (2007) | Full-time employees (*265*)  Age_M_ = 36.3 (6.18) [25-45] | X | .41** | Schwall (2008) | Community adults (*N* = 331)  Age_M_ = 47.2 (10.6) | X | .32** |
| Hershey & Mowen (2000)^a^ | Community adults (*230*)  Age_M_ = 62.6 (12.5) | X | .50** |  |  |  |  |
| Noone et al (2010)^a, d,^ | Community adults (*N* = 1,532)  Age_M_ = 53.4 (2.89) [49-60] | X | .10 - .37** |  |  |  |  |
| Jacobs-Lawson & Hershey (2005)^a^ | Full-time employees (*N* = 265)  Age_M_ = 36.2 (6.18) [25-45] | X | .26** |  |  |  |  |
| **Intended Retirement** | | | | | | | |
| **Study** | **Sample (*N*)** | **Design** | **ES *r*** | **Study** | **Sample (*N*)** | **Design** | **ES *r*** |
| Noone et al (2010)^a, c^ | Community adults (*N* = 1,532)  Age_M_ = 53.4 (2.89) [49-60] | X | -.25** | Schwall (2008) | Community adults (*N* = 233)  Age_M_ = 47 (10.6) | X | .26** |
|  |  |  |  | Schwall (2008) | Community adults (*N* = 387)  Age_M_ = 52 (7.2) [>40] | X | .11* |
|  |  |  |  | Schwall (2008) | Community adults > 40 (*N* = 331)  Age_M_ = 47.2 (10.6) | X | .28** |
|  |  |  |  | Schwall (2008) | Community adults > 40 (*N* = 368)  Age_M_ = 48.6 (10.3) | X | .09* |
|  |  |  |  | Kooij (2010)  Bal et al. (2015) | University workers (*N* = 662)  Age_M_ = 44.2 (10.9)  Dutch taxi drivers (*N* = 168)  Age_M_ = 54.76 (9.02) | X^b^  X | -.04  -.19^*^ |
| *Note.* Standard deviations in parentheses, age inclusion criteria inside brackets. ^a^ originally conceptualized instrument, ^b^ cross-sectional analysis of longitudinal data, ^c^ age-adjusted, ^d^ range across four domains, ^e^ extreme-groups, ^f^ cross-national sample. X = cross-sectional. * *p* < .05, **, *p* < .01. | | | | | | | |
